# Characterization of External Mucosal Microbiomes of Nile Tilapia and Grey Mullet Co-cultured in Semi-Intensive Pond Systems

**DOI:** 10.3389/fmicb.2021.773860

**Published:** 2021-12-13

**Authors:** Ahmed Elsheshtawy, Benjamin Gregory James Clokie, Amaya Albalat, Allan Beveridge, Ahmad Hamza, Abdelaziz Ibrahim, Simon MacKenzie

**Affiliations:** ^1^Institute of Aquaculture, University of Stirling, Stirling, United Kingdom; ^2^Faculty of Aquatic and Fisheries Sciences, Kafrelsheikh University, Kafr El Sheikh, Egypt; ^3^AQUAVET for Fish Nutrition and Health Solutions, Kafr El Sheikh, Egypt; ^4^El Amal Fish Hatchery, Kafr El Sheikh, Egypt

**Keywords:** *Oreochromis niloticus*, *Mugil capito*, gill microbiota, skin microbiota, aquaculture, polyculture

## Abstract

The external mucosal surfaces of the fish harbor complex microbial communities, which may play pivotal roles in the physiological, metabolic, and immunological status of the host. Currently, little is known about the composition and role of these communities, whether they are species and/or tissue specific and whether they reflect their surrounding environment. Co-culture of fish, a common practice in semi-intensive aquaculture, where different fish species cohabit in the same contained environment, is an easily accessible and informative model toward understanding such interactions. This study provides the first in-depth characterization of gill and skin microbiomes in co-cultured Nile tilapia (*Oreochromis niloticus*) and grey mullet (*Mugil capito*) in semi-intensive pond systems in Egypt using 16S rRNA gene-based amplicon sequencing. Results showed that the microbiome composition of the external surfaces of both species and pond water was dominated by the following bacterial phyla: *Proteobacteria, Fusobacteriota, Firmicutes, Planctomycetota, Verrucomicrobiota, Bacteroidota*, and *Actinobacteriota*. However, water microbial communities had the highest abundance and richness and significantly diverged from the external microbiome of both species; thus, the external autochthonous communities are not a passive reflection of their allochthonous communities. The autochthonous bacterial communities of the skin were distinct from those of the gill in both species, indicating that the external microbiome is likely organ specific. However, gill autochthonous communities were clearly species specific, whereas skin communities showed higher commonalities between both species. Core microbiome analysis identified the presence of shared core taxa between both species and pond water in addition to organ-specific taxa within and between the core community of each species. These core taxa included possibly beneficial genera such as *Uncultured Pirellulaceae*, *Exiguobacterium*, and *Cetobacterium* and opportunistic potential pathogens such as *Aeromonas*, *Plesiomonas*, and *Vibrio*. This study provides the first in-depth mapping of bacterial communities in this semi-intensive system that in turn provides a foundation for further studies toward enhancing the health and welfare of these cultured fish and ensuring sustainability.

## Introduction

The external surfaces of fish are considered the first and foremost line of immunological defense, owing to their intimate contact with the external aquatic milieu. These surfaces provide essential protective, respiratory, excretory, sensory, and osmoregulatory functions (Legrand et al., 2018). The skin is the first anatomical and physiological barrier tackling numerous external hazards, and gills function as a selective barrier between the fish and the external environment. Skin and gill surfaces are covered with mucus, which is continuously secreted by goblet cells. This mucus layer is an active immunological barrier consisting of mucins, lysozyme, proteases, antimicrobial peptides, lectins, proteins, and immunoglobulins (Esteban, 2012). Interestingly, these mucosal surfaces are colonized by a highly diverse commensal microbial community, the microbiome (Merrifield and Rodiles, 2015). The microbiome is understood to play a fundamental role in maintaining overall fish health and is likely impacted through host-dependent regulatory pathways and environmental interaction. These mucosal communities may protect the host against pathogenic bacteria through competitive exclusion and mucus homeostasis, facilitating waste product excretion and thus improving host mucosal immunity (Lee and Mazmanian, 2010; van Kessel et al., 2016). Although there appears to be an acceptance for a key role for these external surface microbial communities in finfish health, little is known about their composition and their relationship with their environment as opposed to the finfish gastrointestinal microbiome (Perry et al., 2020).

Many biotic and abiotic factors interact to influence the diversity and the composition of mucosal microbial communities (Butt and Volkoff, 2019). Recently, researchers have started to tackle the microbial ecology of fish, notably the microbial communities of certain niches on the fish body. These studies suggested that some of the commensal microbial communities are unique to specific organs within the same individual, which could be attributed to different functions (Pratte et al., 2018; Zhang et al., 2019; Wang et al., 2020). In parallel, the aquaculture rearing system has been demonstrated to play a key role in the development of the microbiome. Studies have shown that the culture system has a significant impact on the composition of the water microbiome and that changes in water communities were associated with changes in gut microbial communities (Giatsis et al., 2015; Vadstein et al., 2018), suggesting that microbial communities on external surfaces are likely to be more affected by the rearing conditions. Therefore, in addition to tissue-specific approaches, understanding the effect of culture practice on the structure and diversity of the microbiome is critical.

Nile tilapia (*Oreochromis niloticus*) aquaculture has tremendously expanded over the last three decades, and it is currently practiced in >140 countries globally. In terms of production, exceeding 5.8 million tons in 2017 (FAO, 2019), tilapia is the second most-cultured finfish species worldwide. Egypt is one of the top five largest tilapia producers and represents 16.5% of world production. In 2017, tilapia production reached approximately 967,301 tons, representing 66.63% of Egyptian fish production (GAFRD, 2019). Several studies reported high yields of tilapia when raised in polyculture, and this has been linked to the maximum utilization of multiple niches (Tahoun et al., 2013; El-Sayed, 2020). Tilapia have been co-cultured with several fresh and brackish fish species and crustaceans including, striped mullet (*Mugil cephalus*), grey mullet (*Mugil capito*), common carp (*Cyprinus carpio*), silver carp (*Hypophthalmichthys molitrix*), and freshwater prawn (*Macrobrachium rosenbergii*) (Wang and Lu, 2016). In Egypt, integrated tilapia and mullet culture in a semi-intensive system is the most widely used practice. This farming practice has demonstrated various benefits including maximizing the utilization of natural food resources and improving water quality, leading to a more sustainable production system with high commercial returns (El-Sayed, 2020).

Polyculture refers to the co-culture of several non-competing fish species with different ecological requirements and feeding habits. Co-cultured species, by definition, are adapted to similar environments; however, their interactions may be distinct due to their trophic requirements and life cycles. This suggests that distinct microbiome communities may exist in each species that may form a functional host and microbiome relationship. Although some studies have recently investigated the gut microbiome of three Indian major carps (*Labeo rohita*, *Catla catla*, and *Cirrhinus mrigala*) in three polyculture ponds (Mukherjee et al., 2020) and the stomach, intestine, skin, and gill of co-cultured grass carp (*Ctenopharyngodon idella*) and southern catfish (*Silurus meridionalis*) in a laboratory trial (Zhang et al., 2019), to the best of our knowledge, no studies have explored the external surface microbial communities of co-cultured tilapia and mullet and their association within a commercial rearing environment. In this study, semi-intensive culture practice was being used across commercial ponds, where the fish were reared in naturally fertilized ponds with supplemented feed. This study aimed to (1) identify the composition of microbial communities of external surfaces (gill and skin) of Nile tilapia and grey mullet cultured in a semi-intensive system; (2) explore the microbiome signature of the skin and gill for each species; (3) identify the relationship between co-cultured tilapia and mullet surface microbiomes; and (4) explore the relationship between the environment and surface microbial communities under the polyculture practice.

## Materials and Methods

### Ethics Statement

This study was carried out in accordance with the United Kingdom Animal Scientific Procedures Act. The study protocol was approved by the University of Stirling Animal Welfare and Ethical Review Body (AWERB (18/19) 196).

### Fish Samples

*O. niloticus* (*n*=60, 5 farms/12 sample each) and *M. capito* (*n*=24, 2 farms/12 sample each), with an average weight of 226.1 ± 79.83 and 185.7 ± 49.83 g and an average length of 22.08 ± 2.4 and 26.29 ± 2.156 cm, respectively, were randomly collected from five commercial semi-intensive polyculture fish farms located in Kafr Elsheikh province in the Egyptian Nile delta. Fish were placed on ice, and swabs were taken from the gills and skin on site. Samples were stored directly in 1 ml of Longmire’s buffer [0.1 M of Tris, 0.1 M of EDTA, 10 mM of NaCl, and 0.5% (w/v) sodium dodecyl sulfate (SDS)] (Longmire et al., 1997). The five farms all have a semi-intensive polyculture system with pond sizes ranging from 5 to 7 acres. The water source for all the farms was agriculture drainage water, and the water exchange rate was 5% per day. All fish were fed twice per day on commercial extruded floating pellets containing 30% crude protein (Al-Ekhwa Fish Feed, Egypt). The stocking ratio was 5 tilapia:1 mullet (15,000:3,000 fish/acre). The fish were stocked into the farms 6 months before sampling. Because of the difficulty in the sampling of mullet from an open system with low stocking density, mullet were collected from two of the farm sites only. Water samples (6 locations per site/1 L per location) were collected from each farm on the same day of fish sampling and pre-filtered to remove large particles. A two-step filtration using 0.4- and 0.2-μm Whatman Nuclepore filters (GE Healthcare, Chalfont Saint Giles, United Kingdom) was employed to prevent clogging. Filters were stored in 1 ml of Longmire’s buffer until DNA extraction. Water results represent both the 0.2- and 0.4-μm fractions. Water quality parameters for all sites were within the normal range for the two species (temperature 31°C ± 0.5°C, pH 8.2 ± 0.2, salinity 3 ± 0.5 ppt, dissolved oxygen (DO) concentration 5.5 ± 0.5 mg L^–1^ and ammonia 0.07 ± 0.02 mg L^–1^).

### DNA Extraction

DNA was extracted from gill swab, skin swab, and water samples using E.Z.N.A.^®^ Tissue DNA Kit (Omega Bio-Tek Inc., Doraville, GA, United States) according to manufacturer’s protocol, with some modifications. The modifications included a pre-lysis heating of the samples to 95°C for 10 min to increase the efficiency of DNA extraction from gram-positive bacteria and using Longmire’s buffer as a lysis buffer. DNA was eluted from the columns using 100 μl of elution buffer. DNA purity and concentration were evaluated using a NanoDrop ND-1000 Spectrophotometer (Thermo Fisher Scientific, Gloucester, United Kingdom), and concentrations were confirmed using a Qubit 2.0 Fluorometer (Thermo Fisher Scientific, United Kingdom).

### Bacterial 16S rRNA Quantification

A real-time TaqMan absolute qPCR was performed to quantify the bacterial 16S rRNA load in the samples. Primers and FAM-labeled MGB probe targeting the V3–4 region of the bacterial 16S rRNA gene were used (Supplementary Table 1). PCR was performed to amplify the V3–4 region of bacterial 16S rRNA gene of IoA microbiome standard (a mixture of DNA extracted from five bacterial species known to colonize fish: *Aeromonas hydrophila* NCIMB 9240, *Edwardsiella ictaluri* NCIMB 13272, *Pseudomonas aeruginosa* ATCC 27853, *Vibrio anguillarum* NCIMB 6, and *Yersinia ruckeri* NCIMB 2194). The PCR product, a 463-nt fragment from the 16SrRNA of *Y. ruckeri*, was purified using NucleoSpin Gel and PCR Clean-up (Macherey-Nagel, Düren, Germany) according to manufacturer’s protocol. The purified PCR product was ligated into a vector using pGEM^®^-T Easy Vector Systems (Promega, Southampton, United Kingdom) and transformed into XL1-Blue Competent Cells (Agilent Technologies, Santa Clara, CA, United States) following the manufacturer’s protocol. The plasmid DNA was extracted using NucleoSpin Plasmid Quick pure (Macherey-Nagel, Germany), and copy numbers per μl of plasmid DNA were calculated and used for generating a standard curve for absolute quantification. qPCR was performed for all samples, 20 ng/reaction, in triplicate using SensiFAST Probe Lo-ROX Mix (Bioline, London, United Kingdom) with the following conditions: 95°C for 10 min, followed by 40 cycles of amplification (95°C for 30 s and 60°C for 1 min). All qPCR runs showed good linearity (*R*^2^ = 0.989–1, *p* < 0.05) and amplification efficiency of 94.2–102%.

### Bacterial 16S rRNA Amplicon Sequencing

To prepare comparable 16S rRNA microbiome libraries, template DNA used to build the amplicon libraries was normalized to an equal 16S rRNA concentration according to the qPCR assay results. All libraries were constructed using 1 × 10^6^ 16S rRNA copy numbers from each DNA template that ranged in total DNA concentration from 0.62 to 42 ng, and the median value was 7.15 (max. input into the assay was 4.2 μl). Bacterial 16S rRNA Illumina amplicon libraries were generated using a two-step PCR amplicon assay from all the experimental samples, negative sequencing control (NSC), and no template control (NTC) in addition to an IoA microbiome standard. The V4 region of the bacterial 16S rRNA gene was PCR-amplified using 341F and 805R primers overhung with Illumina adaptors and spacers (Supplementary Table 1). A PCR volume of 10 μl comprised 2× NEBNext Ultra II Q5 (New England Biolabs, Hitchin, United Kingdom), 0.2 μM of forward and reverse primer cocktail, and 1 × 10^6^ 16S rRNA copy numbers from DNA templates. The PCR conditions were as follows: an initial denaturation at 98°C for 2 min, 25 cycles of denaturation at 98°C for 15 s, annealing at 54°C for 30 s, and extension at 65°C for 45 s, followed by a final extension step at 65°C for 10 min. PCRs were performed in triplicate before being pooled for PCR 2. Products were examined using 1.5% agarose gel to ensure the correct product size (∼312 bp). Amplicons were purified using AxyPrep Mag PCR Clean-up kit (Axygen Biosciences, Union City, CA, United States) following the manufacturer’s protocol with a modified 1:1 volume of PCR product to AxyPrep beads. Amplicons were eluted into 15 μl of EB buffer (Qiagen, Hilden, Germany). Purified first PCR products were barcoded by the addition of unique index sequences to the 5’ and 3’ ends of each sample using Nextera XT Index Kit (Illumina, San Diego, CA, United States). The indexing PCR was performed with the same conditions as the first PCR for eight cycles. Indexed PCR products were examined using 1.5% agarose gel electrophoresis (∼381 bp), purified using AxyPrep Mag PCR Clean-up kit, and then quantified using Qubit™ dsDNA HS Assay Kit (Thermo Fisher Scientific, Waltham, MA, United States) following the manufacturer’s protocol. An equimolar final pool was prepared from the samples, and sequencing was performed by Novogene (Cambridge, United Kingdom) at PE250 using an S4 flowcell on an Illumina Novaseq (Illumina, United States).

### Bioinformatics and Data Analysis

The raw sequence data provided by Novogene contained 225 paired fastq files. All data processing was performed on a 32 processor HP workstation running Debian Linux (version 10). Sample sequence data (fastq files) were processed (sequence cleaning, clustering in operational taxonomic units (OTUs), and taxonomical classifications) by developing an automated python pipeline using Mothur’s SOP (Schloss Patrick et al., 2009) and the SILVA reference database. To facilitate the high-throughput analysis of multiple sample sets, each containing dozens of fastq files, the pipeline was divided into a set of discrete tasks. Each task was then executed sequentially by running Mothur in batch mode. Scripts containing a set of specific Mothur commands for each task were generated by running a python program that generates the required Mothur commands for each set of sample fastq and corresponding mock fastq files. The total number of the retrieved raw reads was approximately 75.3 million, and the number of sequences per sample ranged between 115,721 and 923,330 with an average of 335,035.8 reads. All statistical analyses were performed in R studio (Version 1.2.5042). Alpha-diversity indices were calculated using the Phyloseq package (McMurdie and Holmes, 2013). The Shapiro–Wilk test was used to verify homogeneity of variance of the alpha-diversity estimates before testing the differences between groups. When the data were normally distributed, alpha-diversity metrics were analyzed using one-way ANOVA and further pairwise comparisons using *t*-test. On the other hand, if the data were not normally distributed, the Kruskal–Wallis test was used, and further pairwise comparisons were performed using a Wilcoxon test (rank-sum test), and *p*-values were adjusted using the Benjamini and Hochberg (BH) correction (Benjamini and Hochberg, 1995). Statistical analysis was conducted with the rstatix package (Kassambara, 2020b). Beta-diversity comparisons were calculated using the Bray–Curtis pairwise distances in packages vegan (Oksanen et al., 2013) and Phyloseq and visualized using non-metric multidimensional scaling (NMDS). Differences between groups were calculated using non-parametric permutational multivariate ANOVA (PERMANOVA) of 1,002 permutations with vegan package. Differences between groups were considered statistically significant at adjusted *p* < 0.05. All figures were produced using the R package ggpubr and ggplot2 (Wickham, 2016; Kassambara, 2020a). In order to compare the relative abundance of taxa between different groups, we generated differential heat trees using Metacoder R package (Foster et al., 2017). The trees illustrate the log2 fold change in taxa abundance. A Wilcoxon rank-sum test followed by a BH [false discovery rate (FDR)] correction was applied to test the differences between the same taxa in different groups, and the *p*-value was set to 0.05. Core microbiome and shared communities were calculated using ampvis2 package (Andersen et al., 2018). The core communities were defined as OTUs that are observed and abundant (belonging to the top 80% of the reads) in all the farms. The average abundance of each OTU in all the samples from a given farm was summed and divided by the total abundance of all OTUs in that farm. Cumulative OTU read abundance was calculated for each farm, and the OTUs containing the top 80% reads were considered abundant. OTUs were grouped according to the number of farms in which they were observed, as well as the number of farms in which they were abundant. The shared microbial community was calculated using only OTUs with relative abundance of at least 0.1%, and the frequency cut-off was placed at 80% to allow only OTUs found in at least 80% of the samples.

## Results

**FIGURE 1:**
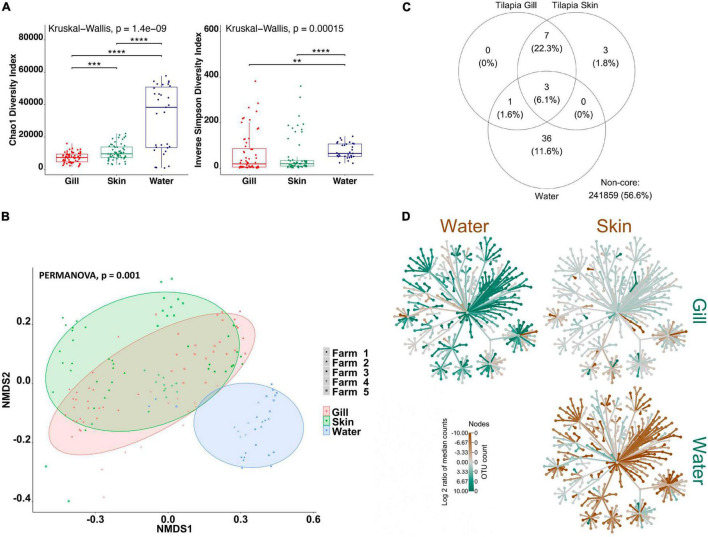
Differential microbial communities in Nile tilapia external surfaces and pond water (*n*=60 fish and *n*=30 water) across five farms. **(A)** Alpha-diversity metrics of tilapia gill and skin microbial communities and pond water. Chao1 and Inverse Simpson were significantly different (Kruskal–Wallis, *p* < 0.0001) in water compared with gill and skin. Dots represent each individual sample, and **p < 0.001, ***p < 0.0001, and ****p < 0.0001. **(B)** Non-metric multidimensional scaling (NMDS) plots based on Bray–Curtis similarity matrix of microbial communities (permutational multivariate ANOVA (PERMANOVA), *p* = 0.001). The colors of the ellipses represent the three groups, and shapes represent the five farms. **(C)** Venn diagram of shared and unique operational taxonomic units (OTUs). The numbers of OTUs with at least 0.1% of relative abundance in 80% of the samples are displayed. Numbers in brackets represent the average relative abundance of the OTUs in that group. **(D)** Differential heat tree matrix showing taxa abundance variation between tilapia gill, skin, and water represented in the dataset (RA > 0.01%). The trees illustrate the pairwise comparisons between the three groups. The color of each taxon represents the log-2 ratio of median proportions of reads observed in each group. Only significant differences, Wilcox rank-sum test followed by a Benjamini–Hochberg [false discovery rate (FDR)] correction are colored. Taxa colored green represent enrichment by row (left to right) and brown by column (top to bottom).

**FIGURE 2:**
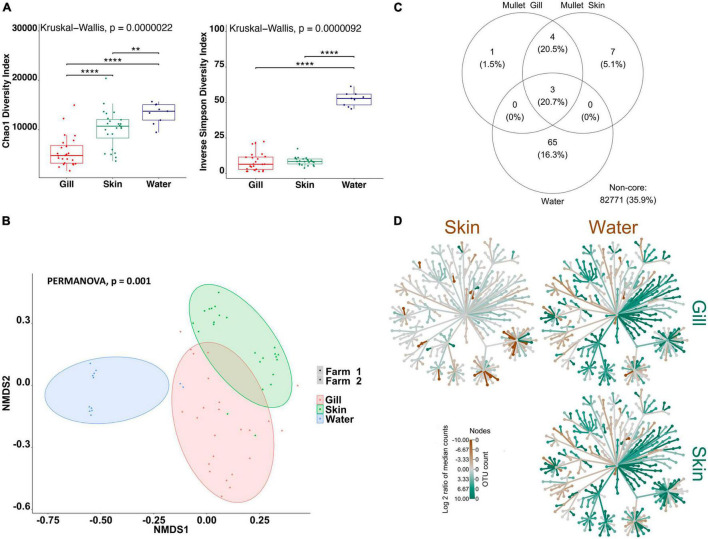
Differential microbial communities in grey mullet external surfaces and pond water (*n*=24 fish and *n*=12 water) across two farms. **(A)** Alpha-diversity metrics of mullet gill and skin microbial communities and pond water. Chao1 and Inverse Simpson were significantly different (Kruskal–Wallis, *p* < 0.00001) in water compared with gill and skin. Dots represent each individual sample, and **p < 0.001 and ****p < 0.0001. **(B)** Non-metric multidimensional scaling (NMDS) plots based on Bray–Curtis similarity matrix of microbial communities [permutational multivariate ANOVA (PERMANOVA), *p* = 0.001]. The colors of the ellipses represent the three groups, and shapes represent the two farms. **(C)** Venn diagram of shared and unique operational taxonomic units (OTUs). The numbers of OTUs with at least 0.1% of relative abundance in 80% of the samples are displayed. Numbers in brackets represent the average relative abundance of the OTUs in that group. **(D)** Differential heat tree matrix showing taxa abundance variation between mullet gill, skin, and water represented in the dataset (RA > 0.01%). The trees illustrate the pairwise comparisons between the three groups. The color of each taxon represents the log-2 ratio of median proportions of reads observed in each group. Only significant differences, Wilcox rank-sum test followed by a Benjamini–Hochberg [false discovery rate (FDR)] correction are colored. Taxa colored green represent enrichment by row (left to right) and brown by column (top to bottom).

Firstly, in order to characterize species-specific differences between gill and skin microbiomes in relation to the water-borne microbial community, we used alpha- and beta-diversity indices for each species separately. In addition, we identified the shared community between the gill, skin, and water for each fish species. Overall, our results indicate that there are significant differences between the gill and skin, in comparison with pond water. Alpha-diversity indices were calculated to evaluate the overall microbial richness, diversity, and evenness within gill, skin, and water samples. A comparison of estimated alpha-diversity indices between the three communities highlighted significant differences in tilapia (Kruskal–Wallis, *p* < 0.0001 for both indices; Figure 1A) and mullet (Kruskal–Wallis, *p* < 0.00001 for both indices; Figure 2A). Bacterial community richness was measured by calculating Chao1, which is a non-parametric estimator of the number of species in a community that gives more weight to low-abundance species (Kim et al., 2017). The pairwise comparisons of Chao1 between tilapia gill, skin, and water revealed that water had the highest microbial richness (Wilcoxon, *p* < 0.00001, Figure 1A). Tilapia skin microbiome had a significantly higher richness than the gill microbiome (Wilcoxon, *p* < 0.001, Figure 1A), and a similar result was observed in mullet, where the water microbial communities had the highest richness (Wilcoxon, *p* < 0.001, Figure 2A) and the skin had higher richness than the gill (Wilcoxon, *p* < 0.0001, Figure 2A). The diversity and evenness of the bacterial community were measured by calculating the Inverse Simpson index, which places greater weight on species’ evenness rather than the richness (Kim et al., 2017). In both species, the comparison of Inverse Simpson index between the gill, skin, and water identified water as having the highest bacterial evenness (Wilcoxon, *p* < 0.001 for both species, Figures 1A, 2A), whereas no significant difference was observed between the gill and skin. Overall, alpha-diversity indices identify a significant difference in bacterial richness and evenness between the external surface microbiome of both species and the rearing pond water.

To determine the variability in microbial communities within and between gill, skin, and water samples, we used the Bray–Curtis dissimilarity index and NMDS to plot the matrix. Significant variations in microbial community were found between the gill, skin, and water of tilapia (PERMANOVA, *p* < 0.001, *R*^2^ = 0.17454; Figure 1B) and mullet (PERMANOVA, *p* < 0.001, *R*^2^ = 0.39335; Figure 2B). Pairwise comparisons, PERMANOVA, revealed that the gill, skin, and water of the two species were significantly diverged (*p* = 0.00099 for all pairwise comparisons). Shared OTUs in tilapia between all experimental samples were calculated (Figure 1C) where shared OTUs between all three groups were represented by three bacterial genera (Supplementary Table 2), whereas seven genera were shared between tilapia gill and skin and one OTU were shared between the gill and water (Supplementary Table 2). On the other hand, three OTUs were identified as shared between mullet gill, skin, and water; and four OTUs were shared between mullet gill and skin (Figure 2C and Supplementary Table 2). The heat trees shown in Figures 1, 2D give a thorough overview of the significantly abundant taxa, when comparing gill, skin, and pond water microbial communities of tilapia (Figure 1D) and mullet (Figure 2D) for taxa with a relative abundance >0.1%. Our results suggest that the external microbial communities of tilapia and mullet are organ specific, and they are unique from the pond water.

**FIGURE 3:**
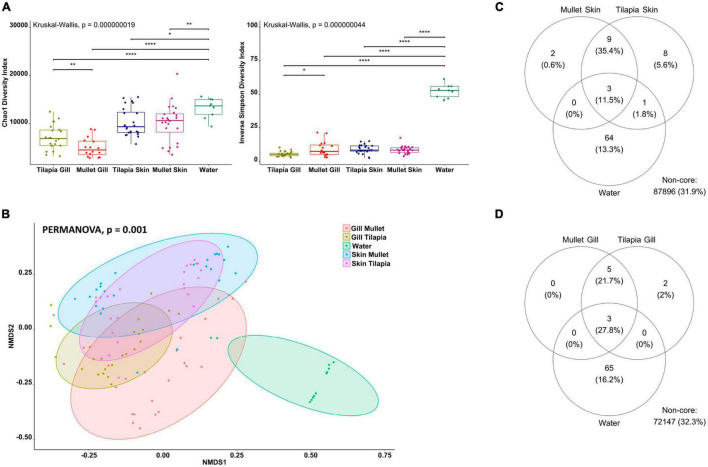
Differential microbial communities in co-cultured Nile tilapia and grey mullet external surfaces and pond water (*n*=24 fish/species and *n*=12 water) across two farms. **(A)** Alpha-diversity metrics of tilapia and mullet gill and skin microbial communities and pond water. Chao1 and Inverse Simpson were significantly different (Kruskal–Wallis, *p* < 0.00000001) in water compared with both species. Dots represent each individual sample, and *p < 0.05, **p < 0.001 and ****p < 0.0001. **(B)** Non-metric multidimensional scaling (NMDS) plots based on Bray–Curtis similarity matrix of microbial communities [permutational multivariate ANOVA (PERMANOVA), *p* = 0.001]. The colors of the ellipses represent the different groups. **(C)** Venn diagram of shared and unique operational taxonomic units (OTUs) between tilapia and mullet gill and water. The numbers of OTUs with at least 0.1% of relative abundance in 80% of the samples are displayed. Numbers in brackets represent the average relative abundance of the OTUs in that group. **(D)** Venn diagram of shared and unique OTUs between tilapia and mullet skin and water.

To explore the impact of the polyculture semi-intensive system on the external microbial communities of the co-cultured species and to characterize inter-species differences, we compared co-cultured Nile tilapia, grey mullet, and rearing water. The alpha diversity of the gill and skin from tilapia and mullet and rearing water microbiota was analyzed using two metrics, Chao1, and Inverse Simpson, reflecting taxonomic richness and evenness. For both alpha-diversity metrics, significant differences were observed between external microbiomes of tilapia and mullet, in comparison with pond water (Kruskal–Wallis, *p* < 0.00001 for both indices; Figure 3A). Tilapia gill showed a significantly higher microbial richness than mullet gill, whereas the mullet gill community had a higher abundance and evenness. On the contrary, there was no significant difference between tilapia and mullet skin communities.

To determine if differences in microbiome structure and composition correlate with fish species, we computed beta diversity using the Bray–Curtis distance. The PERMANOVA of dissimilarity highlighted a significant difference across the microbial communities of the external surfaces of co-cultured tilapia and mullet and rearing water (PERMANOVA, *p* < 0.001, *R*^2^ = 0.41051; Figure 3B). Samples clustered according to species and organ and pairwise comparisons indicated significant differences between the external microbiomes of both species and water (PERMANOVA, *p* < 0.001). However, there was no significant difference between tilapia and mullet skin microbial communities (PERMANOVA, *p* = 0.11). We then identified the shared community between each organ for both species and water (Figures 3C,D). Three genera were identified as the shared OTUs between the gills of the co-cultured species and water (Supplementary Table 2), whereas five genera were shared between tilapia and mullet gill communities (Supplementary Table 2). On the other hand, the shared community between tilapia and mullet skin and water (Figure 3C) displayed three OTUs that were shared between the skin of both species and water; however, nine unique OTUs were shared between the skin of both species. The results indicate that the microbiome is not only organ specific but also species specific. The skin microbiome of the co-cultured tilapia and mullet in the semi-intensive system displayed a shared pattern; however, it was also unique from both the gill and pond water. Interestingly, gill microbial communities of both species appeared to be more selective.

**FIGURE 4:**
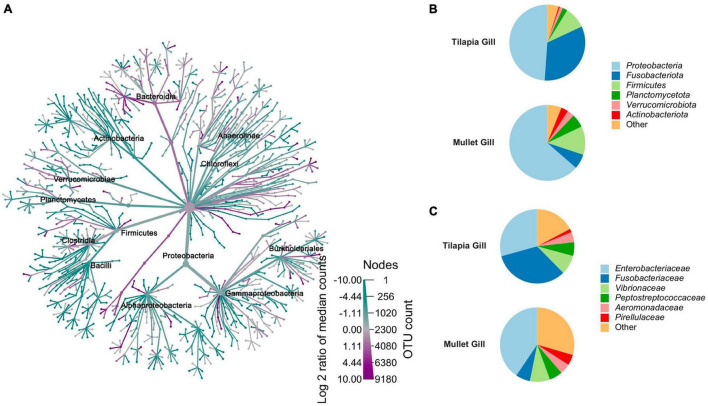
Comparison between co-cultured Nile tilapia and grey mullet gill microbiome composition (*n* = 24 fish/species) across two farms. **(A)** Metacoder heat tree showing the difference in microbiome phylotypes between the tilapia and mullet gill microbial communities. Nodes in the heat tree correspond to phylotypes, as indicated by node labels, while edges link phylotypes in accordance with the taxonomic hierarchy. Node sizes correspond to the number of observed operational taxonomic units (OTUs). Colors represent the log fold difference of a given phylotype’s median relative abundance in tilapia compared with mullet. Only significant differences, Wilcox rank-sum test followed by a Benjamini–Hochberg [false discovery rate (FDR)] correction are colored. Taxa colored dark cyan represent enrichment in mullet and dark magenta in tilapia. **(B)** Pie chart represents mean relative abundance (%) of the most prevalent phyla in tilapia and mullet gill samples; all bacteria with an overall abundance >3% were reported, and bacteria with an abundance of less than 3% were pooled and indicated as “Others”. **(C)** Pie chart represents mean relative abundance (%) of the most prevalent families in tilapia and mullet gill samples; all bacteria with an overall abundance >3% were reported, and bacteria with an abundance of less than 3% were pooled and indicated as “Others”.

The bacterial taxa that were observed across co-cultured tilapia and mullet gill samples were compared to resolve those taxa that vary in association with each species (Figure 4A). *Proteobacteria*, *Actinobacteriota*, *Planctomycetota*, *Fusobacteriota*, and *Verrucomicrobiota* were the dominant phyla in gill samples. The relative abundances of these phyla significantly differed across tilapia and mullet (Figure 4). *Fusobacteriota* manifested higher relative abundance in tilapia gill, whereas *Actinobacteriota*, *Planctomycetota*, *Proteobacteria*, and *Verrucomicrobiota* were significantly more abundant in mullet gill (Figure 4B). Consistently, the family Fusobacteriaceae was more abundant in tilapia gill, while the families Enterobacteriaceae and Pirellulaceae were more abundant in mullet gill (Figure 4C). Our results indicated that the host is more selective for gill microbial communities in this polyculture system.

The gill microbiomes of 60 Nile tilapia collected from five semi-intensive polyculture farms were examined to characterize the structure of gill microbial communities and core microbiome (Supplementary Figure 1). A total of 70 diverse cultured and candidate bacterial phyla were detected from all tilapia gill samples (Supplementary Figure 1A). Gill microbiota was dominated by the members of *Proteobacteria*; abundance on the mean level was 36.18% ± 20.7%. The second most abundant phylum was *Fusobacteriota*, with a mean relative abundance of 18.51% ± 14.77%. The phyla *Firmicutes* (8.44 ± 8.17%), *Planctomycetota* (8.09 ± 6.29%), *Verrucomicrobiota* (5.88 ± 4.85%), *Bacteroidota* (4.28 ± 3.7%), Chloroflexi (4.12 ± 4.18%), *Actinobacteriota* (2.58 ± 2.24%), Desulfobacterota (2.26 ± 2.58%), Acidobacteriota (1.09 ± 1.32%), and Gemmatimonadota (1.01 ± 1.38%) were also abundant in the gill samples (Supplementary Figure 1A). The core microbial taxa were defined as OTUs that are observed in all farms and abundant (belonging to the top 80% of the reads) in all farms. The core gill microbiome of tilapia (constituted 42% of the total reads in farms) comprised six phyla, 10 families, and 11 genera (Supplementary Table 3 and Supplementary Figure 1B). Of the six core phyla, *Proteobacteria* (45%) and *Firmicutes* (18) were the most abundant from all samples. The genera *Unclassified* Peptostreptococcaceae, *Paraclostridium*, *Plesiomonas*, and Burkholderiales *unclassified* were only found in the gills. For the tilapia skin microbiome, we examined the abundances of taxonomic compositions of the 60 samples described above (Supplementary Figure 2). The skin microbiota in tilapia contained 72 bacterial phyla and was dominated by *Proteobacteria* (37.8 ± 27.7), *Firmicutes* (19.67 ± 18.35), *Fusobacteriota* (12.28 ± 8.56), *Planctomycetota* (6.69 ± 4.61), *Verrucomicrobiota* (5.86 ± 5.33), *Bacteroidota* (3.53 ± 2.9), Chloroflexi (2.82 ± 3.12), *Actinobacteriota* (2.79 ± 2.80), *Unclassified Bacteria* (2.78 ± 1.6), and Desulfobacterota (1.67 ± 2.09) (Supplementary Figure 2A). The tilapia skin core microbiome belonged to six phyla, 10 families, and 10 genera. The most dominant phyla were *Proteobacteria* (40%) and *Firmicutes* (20%) (Supplementary Table 3 and Supplementary Figure 2B). The genera *Exiguobacterium*, *Clostridium sensu stricto 1*, and *Acinetobacter* were exclusive to the skin.

The structure of the grey mullet gill microbiota was characterized using the relative abundance of the taxa identified from the 24 samples. A total of 59 different bacterial phyla were detected from all the gill samples. Mullet gill communities were dominated by *Proteobacteria* (63.62 ± 19.94), *Firmicutes* (12.18 ± 12), *Fusobacteriota* (6.29 ± 8.1), *Planctomycetota* (5.74 ± 3.9), *Actinobacteriota* (3.04 ± 2.34), *Verrucomicrobiota* (3.02 ± 2.5), *Unclassified Bacteria* (1.64 ± 0.9), and Chloroflexi (1.51 ± 1.6) (Supplementary Figure 4A). The core microbiome taxa of the mullet gills belonged to six phyla, 11 families, and 14 genera. *Proteobacteria* and *Firmicutes* were the most abundant phyla across the core taxa with a mean abundance of 35.7% for each (Supplementary Table 3 and Supplementary Figure 4B). A total of 60 different bacterial phyla were recovered from all the mullet skin samples. *Proteobacteria* (56.31 ± 23.41), *Firmicutes* (15.67 ± 12.55), *Fusobacteriota* (15.34 ± 12.89), *Planctomycetota* (3.16 ± 2.15), *Verrucomicrobiota* (2.10 ± 2.01), *Unclassified Bacteria* (1.80 ± 1.14), *Bacteroidota* (1.79 ± 1.28), and *Actinobacteriota* (1.50 ± 0.71) were the most abundant phyla retrieved across samples (Supplementary Figure 5A). The core microbiome of the mullet skin consisted of six phyla, 12 families, and 14 genera, with a higher abundance of *Proteobacteria* (57.14%) (Supplementary Table 3 and Supplementary Figure 5B).

The water microbiome of the five farms was composed of 53 different phyla and dominated by the following phyla: *Proteobacteria* (27.27 ± 10), *Planctomycetota* (18.57 ± 4.38), *Verrucomicrobiota* (13.70 ± 6.9), *Actinobacteriota* (13.31 ± 4.6), *Bacteroidota* (11.06 ± 4.69), *Unclassified Bacteria* (6.31 ± 3.04), *Bdellovibrionota* (1.65 ± 0.90), *Firmicutes* (1.57 ± 2.4), Dependentiae (1.15 ± 2.47), and Gemmatimonadota (1.02 ± 0.84) (Supplementary Figure 3A). The core microbiome of water consisted of six phyla (*Proteobacteria* (28%), *Actinobacteriota* (26%), *Planctomycetota* (14%), *Bacteroidota* (14%), *Verrucomicrobiota* (14%), and Gemmatimonadota (4%), 37 families, and 50 genera (Supplementary Table 3 and Supplementary Figure 3B).

## Discussion

This study presents the first in-depth characterization of the external microbiome of co-cultured Nile tilapia and grey mullet in a semi-intensive pond system in Egypt. Our results evidence a significant distinction between the bacterial communities of the external microbiome of both species and rearing pond water. In addition, our results highlight the divergence between gill and skin microbial communities, indicating that the external microbiome is organ specific. The findings of our study illustrate that the gill microbial communities of co-cultured tilapia and mullet in a semi-intensive pond system are selective, showing species specificity, whereas the skin microbiome is similar. *Proteobacteria* was the most predominant phylum in water and the external microbial communities of tilapia and mullet. In addition, the phyla *Fusobacteriota*, *Firmicutes*, *Planctomycetota*, *Verrucomicrobiota*, *Bacteroidota*, and *Actinobacteriota* were highly represented across water, gill, and skin of both species. However, in terms of core microbial community analysis, we identified shared taxa between both species in addition to the presence of organ-specific functional taxa within and between the core community of each species.

Unlike terrestrial animals, fish shape their microbiome within the highly diverse aquatic environment (Rajeev et al., 2021). Semi-intensive aquaculture systems rely on natural food sources, supplementary feed, and/or pond fertilization; and the maintenance of system water quality is mostly achieved through regular water exchange (Oddsson, 2020). Thus, this complex ecosystem has the potential to harbor highly diverse microbial communities. Fish microbial communities have two main sources: autochthonous (host mucosal surface associated) and allochthonous (transient microbiota associated with the environment). In this study, shared OTUs were found between pond water and the external microbial communities, and we identified a significant variation between autochthonous (gill and skin) and allochthonous (pond water) communities in both tilapia and mullet. These results are in accordance with previous studies suggesting that the external autochthonous communities are not a passive reflection of their allochthonous communities (Wang et al., 2010; Chiarello et al., 2015; Pratte et al., 2018; Zhang et al., 2019; Ruiz-Rodríguez et al., 2020), suggesting that despite their intimate contact with their immediate environment, fish external surfaces have their own microbiota. Moreover, water microbial communities showed the highest abundance and richness among all the communities, supporting previous observations by Chiarello et al. (2015) and Zhang et al. (2019). Our findings support the observation that autochthonous communities undergo some form of selection and are reduced in comparison with allochthonous communities.

Bacterial communities of the skin were significantly different from those of the gill in both tilapia and mullet. Therefore, it is possible that host-related factors may influence the shaping of autochthonous mucus bacterial assemblages. The skin showed a higher richness than the gills, whereas no significant differences were observed in their abundance. Interestingly, the number of shared OTUs between the gill and skin was higher than that between water, gill, and skin, suggesting organ-driven selection. Seven OTUs were shared between tilapia gill and skin, and four were shared between mullet organs. The same results were observed in poly-cultured *Carassius auratus gibelio* (Gibel carp) and *Megalobrama amblycephala* Yih (Bluntnose black bream) (Wang et al., 2010), 15 reef fish families (Acanthuridae, Balistidae, Blenniidae, Chaetodontidae, Cirrhitidae, Holocentridae, Labridae, Lethrinidae, Lutjanidae, Mullidae, Pomacanthidae, Pomacentridae, Scaridae, Scorpaenidae, and Serranidae) (Pratte et al., 2018), and 13 wild sympatric Mediterranean Teleost Fish species (*Gobius bucchichi*, *Gobius cruentatus*, *Gobius niger*, *Symphodus tinca*, *Scorpaena notata*, *Serranus scriba*, *Diplodus annularis*, *Diplodus vulgaris*, *Oblada melanura*, *Pagellus bogaraveo*, *Pagellus erythrinus*, and *Spicara maena*) (Ruiz-Rodríguez et al., 2020). The organ microbiome signature could be attributed on the one hand to different metabolic and physiological demands in the host and on the other hand to different environmental factors.

Results indicate that there are significant differences between the co-cultured tilapia and mullet external microbiomes, particularly gill microbial communities. The mullet gill microbial communities were more diverse than tilapia; however, the tilapia gill communities had a higher richness. On the contrary and importantly, there were no significant differences between the skin microbial communities of the two species. Nine genera representing 35.4% of the core community were shared between tilapia and mullet skin, whereas five genera (21.7%) were shared between gill communities. In general, host specificity was reported in grass carp and southern catfish under laboratory conditions (Zhang et al., 2019), 13 Wild Sympatric Mediterranean Teleost Fish species (Ruiz-Rodríguez et al., 2020), butterflyfishes (*Chaetodon lunulatus*, *Chaetodon ornatissimus*, *Chaetodon reticulatus*, and *Chaetodon vagabundus*) (Reverter et al., 2017), and poly-cultured gibel carp and bluntnose black bream (Wang et al., 2010). For species-specific skin microbiomes, Larsen et al. (2013) reported variabilities between six species (*M. cephalus*, *Lutjanus campechanus*, *Cynoscion nebulosus*, *Cynoscion arenarius*, *Micropogonias undulatus*, and *Lagodon rhomboides*) sampled from the Gulf of Mexico; and Chiarello et al. (2015) found variabilities in skin microbiota of European seabass (*Dicentrarchus labrax*) and gilthead seabream (*Sparus aurata*). However, in the latter two studies, sampling regimes represented a variety of months at multiple locations with different temperatures and salinities or in two monospecific tanks. In contrast, this study indicated similarities between skin microbiota of co-cultured tilapia and mullet using simultaneous sampling regimes in a commercial semi-intensive culture system. Previous observations in poly-cultured gibel carp and bluntnose black bream raised in a pond system support our current findings highlighting that poly-cultured fish in a pond system may have similarities in their skin microbial communities, although further studies are required to support this observation.

The mucosal surface of the gills is a unique habitat for host-associated microbial communities (Pratte et al., 2018). Gill microbiome selection is likely influenced by a range of abiotic and biotic factors and linked to species-specific life cycles where local concentrations of oxygen and nutrient availability, environmental stress, and gill function among others may play important roles. In the current study, we have characterized the gill microbial communities of two co-cultured species that display different life histories and adaptations. The grey mullet are a catadromous fish that spawn in saltwater, with juveniles being capable of osmoregulation and can tolerate salinity up to 35 ppt; however, most of the life cycle is in freshwater, and wild fry are the main source used to populate farms in Egypt. Conversely, tilapia are mouth breeder, and commercial hatcheries supply the farms with fingerlings. The difference in their lifestyle and origin likely influences the observed differences in microbial community at the level of the gill. Of importance is the consideration that gill microbiomes that are intimately associated with this key organ may contribute to host-specific functionality at a metabolic, physiological, or immunological level. Therefore, further examination of species-specific gill microbiomes and the functional interplay with the host in terms of fitness represents an exciting area for new research.

The observed dynamics of bacterial relative abundance within and between the external microbiome of tilapia and mullet were pronounced. Despite *Proteobacteria* being the most abundant in both species, its abundance significantly varied between fish and pond water. The pond water had the lowest abundance of *Proteobacteria*, whereas it was highly enriched in the mullet gill. The dominance of *Proteobacteria* in the external microbiome of teleost fish has been indicated in previous studies (Chiarello et al., 2015; Legrand et al., 2018; Zhang et al., 2019; Wu et al., 2021). Communities with enriched *Proteobacteria* may reflect the advantage of unique niche colonization for enhancing microbial growth (Zhang et al., 2019) and a significant role in the microbiome mucosal barrier for this bacterial phyla. Interestingly, several opportunistic aquatic pathogens, such as *Aeromonas*, *Vibrio*, and *Plesiomonas*, belong to *Proteobacteria*; and their presence may stimulate and maintain mucosal immunity (Wu et al., 2021). The higher abundance of *Proteobacteria* in external microbial communities was followed by *Fusobacteriota* and *Firmicutes*, which have been reported as ubiquitous phyla in the external microbiome of the fish (Reverter et al., 2017; Zhang et al., 2019; Wu et al., 2021). *Fusobacteriota* was remarkably enriched in tilapia gill, whereas *Firmicutes* was enriched in the skin for both species. *Firmicutes* produces short-chain fatty acids that are essential nutrients to the mucosal cells (Koh et al., 2016). *Fusobacteriota* is well-known to produce butyrate, a short-chain fatty acid that is the end-product of carbohydrate fermentation, including that found in mucins. Butyrate provides many benefits to the host such as enhancing mucus secretion, providing energy supply to host cells, and acting as an anti-inflammatory (von Engelhardt et al., 1998; Larsen et al., 2014). Moreover, butyric acid was reported to inhibit freshwater fish pathogens (Nuez-Ortin et al., 2012), and some members of *Fusobacteriota* are known to produce vitamin B12 (Finegold et al., 2003). The presence of *Fusobacteriota* in the external surface microbiome has been suggested to have a protective role in fish gills (Reverter et al., 2017). Notably, the phyla *Planctomycetota*, *Verrucomicrobiota*, *Bacteroidota*, and *Actinobacteriota* were enriched in the pond water. These phyla were identified in freshwater pond bacterioplankton (Qin et al., 2016; Fan et al., 2017), and a symbiotic relationship was reported between these phyla in the aquatic environment (Kaboré et al., 2020). The higher abundance of these taxa in pond water and the lower abundance in the gill and skin of both fish suggests that these taxa were derived from the allochthonous community, although their lower abundance on the external surfaces of the fish may be due to selective pressures that are organ specific.

The core microbiome of external surfaces of tilapia and mullet and pond water was mainly composed of the phyla *Proteobacteria*, *Firmicutes*, *Fusobacteriota*, *Actinobacteriota*, *Planctomycetota*, *Bacteroidota*, and *Verrucomicrobiota*. These results are consistent with other studies in other teleost fish (Llewellyn et al., 2014). In the present study, the genera Enterobacteriaceae *unclassified*, *Vibrio*, and *LD29* were detected in the core microbiome for all niches. Besides these three genera, *Cetobacterium*, *Aeromonas*, and *Uncultured Pirellulaceae* were also found in the core community of both fish. Several core taxa comprising possibly beneficial, opportunistic, and potentially pathogenic bacteria were recovered from the skin and gill of “healthy” tilapia and mullet. *Uncultured Pirellulaceae* is one such taxon recovered from the gill and skin, and members of the family Pirellulaceae are found in both the fresh and marine water environments (Kellogg et al., 2016; Parata et al., 2020). These bacteria belong to the ammonia-oxidizing bacteria, and their presence in the gill and skin is potentially crucial for the excretion of nitrogenous waste products. The genus *Exiguobacterium* was identified in the core community of the skin in both species. *Exiguobacterium* accommodates many versatile species, and it has been isolated from diverse environments including aquatic environments (Kasana and Pandey, 2018). The genus *Exiguobacterium* possesses various stress-responsive genes, helping them to colonize and thrive in various ecological niches and produce hydrolytic enzymes that could be beneficial for the host (Vishnivetskaya et al., 2009). Strains belonging to *Exiguobacterium* have a large array of functions such as degradation of environmental pollutants, pesticide removal, and algicidal, antifungal, and antibacterial activities (Selvakumar et al., 2009; Shanthakumar et al., 2015; Li et al., 2017). Moreover, Pandey and Bhatt (2015) reported the protective effect of *Exiguobacterium* against arsenic-induced toxicity and oxidative damage in freshwater fish. *Cetobacterium* has been reported as a major component of freshwater fish gut and gill microbial communities (Ramírez et al., 2018). *Cetobacterium* is essential for vitamin B12 production and inhibition of pathogenic bacterial growth, and its abundance in the gut was suggested to be essential for healthy gut microbiota. Among the opportunistic pathogens, *Aeromonas*, *Plesiomonas*, and *Vibrio* were identified within the core microbial taxa; however, they have been previously reported in healthy tilapia gill and gut microbiome (Pakingking et al., 2015; Wu et al., 2021). The genera *Unclassified* Peptostreptococcaceae and *Clostridium sensu stricto 1* observed in the core community were also reported in the core gut microbiome of tilapia (Bereded et al., 2020).

In conclusion, the current study presents the first comprehensive high-throughput characterization of the gill and skin microbiome of co-cultured Nile tilapia and grey mullet in a semi-intensive pond system. Our results highlighted the distinction between the external microbiomes of both species and pond water. Both tilapia and mullet simultaneously exhibited diverse bacterial community signatures between the skin and gill, suggesting the role of different metabolic and physiological activities in shaping the autochthonous mucus bacterial assemblages at the organ scale. The gill microbial communities of co-cultured tilapia and mullet in a semi-intensive pond system diverged between species, whereas skin communities showed similarities across species. Further investigation of species-specific gill microbiomes and the functional interaction with the host is highly recommended. Interestingly, the core microbiome characterization identified beneficial functional genera such as *Uncultured Pirellulaceae*, *Exiguobacterium*, and *Cetobacterium*. This study provides new insights into the external microbiomes of poly-cultured fishes in semi-intensive pond systems that will foster advances in health and welfare management and enhance sustainability and food security in these extensive aquaculture systems.

## Data Availability Statement

Bacterial 16S rRNA Amplicon data: SRA. Accession id; PRJNA770664.

## Ethics Statement

The animal study was reviewed and approved by the University of Stirling Animal Welfare and Ethical Review Body (AWERB (18/19) 196).

## Author Contributions

AE: conceptualization, methodology, validation, formal analysis, investigation, and writing—original draft. BC: conceptualization, methodology, validation, formal analysis, and writing—review and editing. AA: conceptualization, methodology, validation, supervision, and writing—review and editing. AB: software, formal analysis, and data curation. AH: resources. AI: resources. SM: conceptualization, methodology, validation, supervision, funding acquisition, and writing—review and editing. All authors contributed to the article and approved the submitted version.

## Conflict of Interest

The authors declare that the research was conducted in the absence of any commercial or financial relationships that could be construed as a potential conflict of interest.

## Publisher’s Note

All claims expressed in this article are solely those of the authors and do not necessarily represent those of their affiliated organizations, or those of the publisher, the editors and the reviewers. Any product that may be evaluated in this article, or claim that may be made by its manufacturer, is not guaranteed or endorsed by the publisher.

## References

[B1] Andersen K. S. Kirkegaard R. H. Karst S. M. Albertsen M. (2018). ampvis2: an R package to analyse and visualise 16S rRNA amplicon data. *BioRxiv* [Preprint] BioRxiv 299537*, 10.1101/299537

[B2] Benjamini Y. Hochberg Y. (1995). Controlling the false discovery rate: a practical and powerful approach to multiple testing. *J. R. Stat. Soc. Ser. B (Methodological)* 57 289–300. 10.1111/j.2517-6161.1995.tb02031.x

[B3] Bereded N. K. Curto M. Domig K. J. Abebe G. B. Fanta S. W. Waidbacher H. (2020). Metabarcoding analyses of gut microbiota of nile tilapia (*Oreochromis niloticus*) from lake awassa and lake chamo. *Ethiopia. Microorganisms* 8:1040. 10.3390/microorganisms8071040 32668725 PMC7409238

[B4] Butt R. L. Volkoff H. (2019). Gut microbiota and energy homeostasis in fish. *Front. Endocrinol.* 10:9. 10.3389/fendo.2019.00009 30733706 PMC6353785

[B5] Chiarello M. Villéger S. Bouvier C. Bettarel Y. Bouvier T. (2015). High diversity of skin-associated bacterial communities of marine fishes is promoted by their high variability among body parts, individuals and species. *FEMS Microbiol. Ecol.* 91:fiv061. 10.1093/femsec/fiv061 26048284

[B6] El-Sayed A.-F. M. (2020). “Chapter 5 – semi-intensive culture,” in *Tilapia Culture*, 2nd Edn, ed. El-Sayed A.-F. M. (Cambridge, MA: Academic Press), 69–101. 10.1016/B978-0-12-816509-6.00005-7

[B7] Esteban M. Á (2012). An overview of the immunological defenses in fish skin. *ISRN Immunol.* 2012:853470. 10.5402/2012/853470

[B8] Fan L. Chen J. Meng S. Song C. Qiu L. Hu G. (2017). Characterization of microbial communities in intensive GIFT tilapia (*Oreochromis niloticus*) pond systems during the peak period of breeding. *Aquacult. Res.* 48 459–472. 10.1111/are.12894

[B9] FAO (2019). *Global Aquaculture Production 1950-2016.* Rome: Food and Agriculture Organization of the United Nations.

[B10] Finegold S. M. Vaisanen M.-L. Molitoris D. R. Tomzynski T. J. Song Y. Liu C. (2003). *Cetobacterium somerae* sp. nov. from human feces and emended description of the genus *Cetobacterium*. Syst. Appl. Microbiol. 26 177–181. 10.1078/072320203322346010 12866843

[B11] Foster Z. S. Sharpton T. J. Grünwald N. J. (2017). Metacoder: an R package for visualization and manipulation of community taxonomic diversity data. *PLoS Comp. Biol.* 13:e1005404. 10.1371/journal.pcbi.1005404 28222096 PMC5340466

[B12] GAFRD (2019). *Egyptian Fish Statistics Yearbook 2017 Cairo, Egypt: General Authority for Fish Resources Development, Ministry of Agriculture.* Rome: FAO.

[B13] Giatsis C. Sipkema D. Smidt H. Heilig H. Benvenuti G. Verreth J. (2015). The impact of rearing environment on the development of gut microbiota in tilapia larvae. *Sci. Rep.* 5:18206. 10.1038/srep18206 26658351 PMC4676014

[B14] Kaboré O. D. Godreuil S. Drancourt M. (2020). Planctomycetes as host-associated bacteria: a perspective that holds promise for their future isolations, by mimicking their native environmental niches in clinical microbiology laboratories. *Front. Cell. Infect. Microbiol.* 10:519301. 10.3389/fcimb.2020.519301 33330115 PMC7734314

[B15] Kasana R. C. Pandey C. B. (2018). Exiguobacterium: an overview of a versatile genus with potential in industry and agriculture. *Crit. Rev. Biotechnol.* 38 141–156. 10.1080/07388551.2017.1312273 28395514

[B16] Kassambara A. (2020b). *RSTATIX. Pipe-Friendly Framework for Basic Statistical Test.* Available online at: https://rpkgs.datanovia.com/rstatix/

[B17] Kassambara A. (2020a). *ggplot2. Based Publication Ready Plots [R package ggpubr version 0.2. 5].*

[B18] Kellogg C. A. Ross S. W. Brooke S. D. (2016). Bacterial community diversity of the deep-sea octocoral *Paramuricea placomus*. *PeerJ* 4:e2529. 10.7717/peerj.2529 27703865 PMC5047221

[B19] Kim B. R. Shin J. Guevarra R. Lee J. H. Kim D. W. Seol K. H. (2017). Deciphering diversity indices for a better understanding of microbial communities. *J Microbiol. Biotechnol.* 27 2089–2093. 10.4014/jmb.1709.09027 29032640

[B20] Koh A. De Vadder F. Kovatcheva-Datchary P. Bäckhed F. (2016). From dietary fiber to host physiology: short-chain fatty acids as key bacterial metabolites. *Cell* 165 1332–1345. 10.1016/j.cell.2016.05.041 27259147

[B21] Larsen A. Tao Z. Bullard S. A. Arias C. R. (2013). Diversity of the skin microbiota of fishes: evidence for host species specificity. *FEMS Microbiol. Ecol.* 85 483–494. 10.1111/1574-6941.12136 23607777

[B22] Larsen A. M. Mohammed H. H. Arias C. R. (2014). Characterization of the gut microbiota of three commercially valuable warmwater fish species. *J. Appl. Microbiol.* 116 1396–1404. 10.1111/jam.12475 24529218

[B23] Lee Y. K. Mazmanian S. K. (2010). Has the microbiota played a critical role in the evolution of the adaptive immune system? *Science (New York, N.Y.)* 330 1768–1773. 10.1126/science.1195568 21205662 PMC3159383

[B24] Legrand T. P. R. A. Catalano S. R. Wos-Oxley M. L. Stephens F. Landos M. Bansemer M. S. (2018). the inner workings of the outer surface: skin and gill microbiota as indicators of changing gut health in yellowtail kingfish. *Front. Microbiol.* 8:2664. 10.3389/fmicb.2017.02664 29379473 PMC5775239

[B25] Li Y. Liu L. Xu Y. Li P. Zhang K. Jiang X. (2017). Stress of algicidal substances from a bacterium *Exiguobacterium* sp. h10 on *Microcystis aeruginosa*. *Lett. Appl. Microbiol.* 64 57–65. 10.1111/lam.12678 27714825

[B26] Llewellyn M. S. Boutin S. Hoseinifar S. H. Derome N. (2014). Teleost microbiomes: the state of the art in their characterization, manipulation and importance in aquaculture and fisheries. *Front. Microbiol.* 5:207. 10.3389/fmicb.2014.00207 24917852 PMC4040438

[B27] Longmire J. L. Maltbie M. Baker R. J. (1997). *Use of” Lysis Buffer” in DNA Isolation and its Implication for Museum Collections.* Lubbock, TX: Museum of Texas Tech University. 10.5962/bhl.title.143318

[B28] McMurdie P. J. Holmes S. (2013). phyloseq: an r package for reproducible interactive analysis and graphics of microbiome census data. *PLoS One* 8:e61217. 10.1371/journal.pone.0061217 23630581 PMC3632530

[B29] Merrifield D. L. Rodiles A. (2015). “10 - The fish microbiome and its interactions with mucosal tissues,” in *Mucosal Health in Aquaculture*, eds Beck B. H. Peatman E. (San Diego, CA: Academic Press), 273–295. 10.1016/B978-0-12-417186-2.00010-8

[B30] Mukherjee A. Rodiles A. Merrifield D. L. Chandra G. Ghosh K. (2020). Exploring intestinal microbiome composition in three Indian major carps under polyculture system: a high-throughput sequencing based approach. *Aquaculture* 524:735206. 10.1016/j.aquaculture.2020.735206

[B31] Nuez-Ortin W. Prado S. Toranzo A. (2012). “Antimicrobial properties of butyric acid and other organic acids against pathogenic bacteria affecting the main aquatic species,” in *Proceedings of the Conference proceedings Aqua Conference 2012*, (Prague).

[B32] Oddsson G. V. (2020). A definition of aquaculture intensity based on production functions—The aquaculture production intensity scale (APIS). *Water* 12:765. 10.3390/w12030765

[B33] Oksanen J. Blanchet F. G. Kindt R. Legendre P. Minchin P. R. O’hara R. (2013). Package ‘vegan’. *Commun. Ecol. Package Version* 2 1–295.

[B34] Pakingking R. Palma P. Usero R. (2015). Quantitative and qualitative analyses of the bacterial microbiota of tilapia (*Oreochromis niloticus*) cultured in earthen ponds in the Philippines. *World J. Microbiol. Biotechnol.* 31 265–275. 10.1007/s11274-014-1758-1 25555375

[B35] Pandey N. Bhatt R. (2015). *Exiguobacterium* mediated arsenic removal and its protective effect against arsenic induced toxicity and oxidative damage in freshwater fish *Channa striata*. *Toxicol. Rep.* 2 1367–1375. 10.1016/j.toxrep.2015.10.002 28962479 PMC5598528

[B36] Parata L. Mazumder D. Sammut J. Egan S. (2020). Diet type influences the gut microbiome and nutrient assimilation of genetically improved farmed tilapia (*Oreochromis niloticus*). *PLoS One* 15:e0237775. 10.1371/journal.pone.0237775 32813739 PMC7446784

[B37] Perry W. B. Lindsay E. Payne C. J. Brodie C. Kazlauskaite R. (2020). The role of the gut microbiome in sustainable teleost aquaculture. *Proc. R. Soc. B Biol. Sci.* 287:20200184. 10.1098/rspb.2020.0184 32372688 PMC7282919

[B38] Pratte Z. A. Besson M. Hollman R. D. Stewart F. J. (2018). The gills of reef fish support a distinct microbiome influenced by host-specific factors. *Appl. Environ. Microbiol.* 84 e00063–18. 10.1128/AEM.00063-18 29453266 PMC5930318

[B39] Qin Y. Hou J. Deng M. Liu Q. Wu C. Ji Y. (2016). Bacterial abundance and diversity in pond water supplied with different feeds. *Sci. Rep.* 6:35232. 10.1038/srep35232 27759010 PMC5069485

[B40] Rajeev R. Adithya K. K. Kiran G. S. Selvin J. (2021). Healthy microbiome: a key to successful and sustainable shrimp aquaculture. *Rev. Aquacult.* 13 238–258. 10.1111/raq.12471

[B41] Ramírez C. Coronado J. Silva A. Romero J. (2018). cetobacterium is a major component of the microbiome of giant amazonian fish (*Arapaima gigas*) in Ecuador. *Animals* 8:189. 10.3390/ani8110189 30352962 PMC6262583

[B42] Reverter M. Sasal P. Tapissier-Bontemps N. Lecchini D. Suzuki M. (2017). Characterisation of the gill mucosal bacterial communities of four butterflyfish species: a reservoir of bacterial diversity in coral reef ecosystems. *FEMS Microbiol. Ecol.* 93:fix051. 10.1093/femsec/fix051 28431143

[B43] Ruiz-Rodríguez M. Scheifler M. Sanchez-Brosseau S. Magnanou E. West N. Suzuki M. (2020). Host species and body site explain the variation in the microbiota associated to wild sympatric mediterranean teleost fishes. *Microb. Ecol.* 80 212–222. 10.1007/s00248-020-01484-y 31932881

[B44] Schloss Patrick D. Westcott Sarah L. Ryabin T. Hall Justine R. Hartmann M. Hollister Emily B. (2009). Introducing mothur: open-source, platform-independent, community-supported software for describing and comparing microbial communities. *Appl. Environ. Microbiol.* 75 7537–7541. 10.1128/AEM.01541-09 19801464 PMC2786419

[B45] Selvakumar G. Joshi P. Nazim S. Mishra P. K. Kundu S. Gupta H. S. (2009). Exiguobacterium acetylicum strain 1P (MTCC 8707) a novel bacterial antagonist from the North Western Indian Himalayas. *World J. Microbiol. Biotechnol.* 25 131–137. 10.1007/s11274-008-9874-4

[B46] Shanthakumar S. Duraisamy P. Vishwanath G. Selvanesan B. C. Ramaraj V. David B. V. (2015). Broad spectrum antimicrobial compounds from the bacterium *Exiguobacterium mexicanum* MSSRFS9. *Microbiol. Res.* 178 59–65. 10.1016/j.micres.2015.06.007 26302848

[B47] Tahoun A.-A. Suloma A. Hammouda Y. Abo-State H. El-Haroun E. (2013). The effect of stocking different ratios of nile tilapia *Oreochromis niloticus*, striped mullet *Mugil cephalus*, and thinlip grey mullet liza ramada in polyculture ponds on biomass yield, feed efficiency, and production economics. *North Am. J. Aquacult.* 75 548–555. 10.1080/15222055.2013.826764

[B48] Vadstein O. Attramadal K. J. K. Bakke I. Forberg T. Olsen Y. Verdegem M. (2018). Managing the microbial community of marine fish larvae: a holistic perspective for larviculture. *Front. Microbiol.* 9:1820. 10.3389/fmicb.2018.01820 30210457 PMC6119882

[B49] van Kessel M. A. H. J. Mesman R. J. Arshad A. Metz J. R. Spanings F. A. T. van Dalen S. C. M. (2016). Branchial nitrogen cycle symbionts can remove ammonia in fish gills. *Environ. Microbiol. Rep.* 8 590–594. 10.1111/1758-2229.12407 27040730

[B50] Vishnivetskaya T. A. Kathariou S. Tiedje J. M. (2009). The *Exiguobacterium* genus: biodiversity and biogeography. *Extremophiles* 13 541–555. 10.1007/s00792-009-0243-5 19381755

[B51] von Engelhardt W. Bartels J. Kirschberger S. Meyer zu Düttingdorf H. D. Busche R. (1998). Role of short-chain fatty acids in the hind gut. *Vet. Q.* 20 (Suppl 3) S52–S59. 10.1080/01652176.1998.9694970 9689727

[B52] Wang M. Lu M. (2016). Tilapia polyculture: a global review. *Aquacult. Res.* 47 2363–2374. 10.1111/are.12708

[B53] Wang M. Yi M. Lu M. Gao F. Liu Z. Huang Q. (2020). Effects of probiotics Bacillus cereus NY5 and *Alcaligenes faecalis* Y311 used as water additives on the microbiota and immune enzyme activities in three mucosal tissues in Nile tilapia *Oreochromis niloticus* reared in outdoor tanks. *Aquacult. Rep.* 17:100309. 10.1016/j.aqrep.2020.100309

[B54] Wang W. Zhou Z. He S. Liu Y. Cao Y. Shi P. (2010). Identification of the adherent microbiota on the gills and skin of poly-cultured gibel carp (*Carassius auratus gibelio*) and bluntnose black bream (*Megalobrama amblycephala* Yih). *Aquacult. Res.* 41 e72–e83. 10.1111/j.1365-2109.2009.02459.x

[B55] Wickham H. (2016). “Programming with ggplot2,” in *ggplot2: Elegant Graphics for Data Analysis*, ed. Wickham H. (Cham: Springer International Publishing), 241–253. 10.1007/978-3-319-24277-4_12

[B56] Wu Z. Zhang Q. Lin Y. Hao J. Wang S. Zhang J. (2021). Taxonomic and functional characteristics of the gill and gastrointestinal microbiota and its correlation with intestinal metabolites in NEW GIFT strain of farmed adult nile tilapia (*Oreochromis niloticus*). *Microorganisms* 9:617. 10.3390/microorganisms9030617 33802740 PMC8002438

[B57] Zhang Z. Li D. Xu W. Tang R. Li L. (2019). Microbiome of co-cultured fish exhibits host selection and niche differentiation at the organ scale. *Front. Microbiol.s* 10:2576. 10.3389/fmicb.2019.02576 31781072 PMC6856212

