# Supplementary material for: Characterization of External Mucosal Microbiomes of Nile Tilapia and Grey Mullet Co-cultured in Semi-Intensive Pond Systems
Source: Front Microbiol. 2021 Dec 13;12:773860. doi: 10.3389/fmicb.2021.773860 (PMC8710667; doi:10.3389/fmicb.2021.773860)
Supplement: Supplementary file 1 [file Table_1.docx]

**List of Supplementary Tables**

**Supplementary Table 1. List of the primers and probe used in the study**

| Primer | Primer Sequence (5'- 3') | Target | Product size |
| --- | --- | --- | --- |
| V3-4 [314F] | CCTACGGGNGGCWGCAG | 16S rRNA  V3-4 | ~466bp |
| V3-4 [805R] | GACTACHVGGGTATCTAATCC |  |  |
| V3 V4 TaqMan | ATTACCGCGGCTGCTGG |  |  |
| V4 Sp4 Mix F | TCGTCGGCAGCGTCAGATGTGTATAAGAGACAGAGTCAYTGGGYDTAAAGN*G | 16S rRNA V4 | ~245bp |
| V4 Sp4 Mix R1 | GTCTCGTGGGCTCGGAGATGTGTATAAGAGACAGCTACCRGGGTHTCTAATC*C |  |  |
| V4 Sp4 Mix R2 | GTCTCGTGGGCTCGGAGATGTGTATAAGAGACAGCTACCAGAGTATCTAATT*C |  |  |
| V4 Sp4 Mix R3 | GTCTCGTGGGCTCGGAGATGTGTATAAGAGACAGCCTACDSRGGTMTCTAAT*C |  |  |
| V4 Sp4 Mix R4 | GTCTCGTGGGCTCGGAGATGTGTATAAGAGACAGCTACNVGGGTATCTAAT*C |  |  |

**Supplementary Table 2. List of the shared microbiota**

| Species- Organ | Taxonomy |
| --- | --- |
| Tilapia Gill  Tilapia Skin  water | Bacteria; *Planctomycetota; Planctomycetes; Pirellulales; Pirellulaceae; uncultured* |
|  | Bacteria; *Verrucomicrobiota; Verrucomicrobiae; Chthoniobacterales; Chthoniobacteraceae; LD29* |
|  | Bacteria; *Planctomycetota; Phycisphaerae; Phycisphaerales; Phycisphaeraceae; Phycisphaeraceae unclassified* |
| Tilapia Gill  Tilapia Skin | Bacteria; *Proteobacteria; Gammaproteobacteria; Enterobacterales; Enterobacteriaceae; Enterobacteriaceae unclassified* |
|  | Bacteria; *Firmicutes; Clostridia; Clostridiales; Clostridiaceae; Clostridium sensu stricto 1* |
|  | Bacteria; *Proteobacteria; Gammaproteobacteria; Aeromonadales; Aeromonadaceae; Aeromonas* |
|  | Bacteria; *Fusobacteriota; Fusobacteriia; Fusobacteriales; Fusobacteriaceae; Cetobacterium* |
|  | Bacteria; *Firmicutes; Clostridia; Clostridiales; Clostridiaceae; Clostridiaceae unclassified* |
|  | Bacteria; *Firmicutes; Clostridia; Peptostreptococcales-Tissierellales; Peptostreptococcaceae; Peptostreptococcaceae unclassified* |
|  | Bacteria; *Bacteroidota; Bacteroidia; Bacteroidales; Barnesiellaceae; uncultured* |
| Tilapia Gill  Water | Bacteria; *Planctomycetota; Phycisphaerae; Phycisphaerales; Phycisphaeraceae; Phycisphaeraceae unclassified* |
| Mullet Gill  Mullet Skin  Water | Bacteria; *Proteobacteria; Gammaproteobacteria; Enterobacterales; Enterobacteriaceae; Enterobacteriaceae unclassified* |
|  | Bacteria; *Planctomycetota; Planctomycetes; Pirellulales; Pirellulaceae; uncultured* |
|  | Bacteria; *Verrucomicrobiota; Verrucomicrobiae; Chthoniobacterales; Chthoniobacteraceae; LD29* |
| Mullet Gill  Mullet Skin | Bacteria; *Fusobacteriota; Fusobacteriia; Fusobacteriales*; *Fusobacteriaceae; Cetobacterium* |
|  | Bacteria; *Proteobacteria; Gammaproteobacteria; Vibrionales; Vibrionaceae; Vibrio* |
|  | Bacteria; *Proteobacteria; Gammaproteobacteria; Aeromonadales; Aeromonadaceae; Aeromonas* |
|  | Bacteria; *Proteobacteria; Gammaproteobacteria; Enterobacterales; Enterobacteriaceae; Plesiomonas* |
| Tilapia Gill  Mullet Gill  Water | Bacteria; *Proteobacteria; Gammaproteobacteria; Enterobacterales; Enterobacteriaceae; Enterobacteriaceae unclassified* |
|  | Bacteria; *Planctomycetota; Planctomycetes; Pirellulales; Pirellulaceae; uncultured* |
|  | Bacteria; *Verrucomicrobiota; Verrucomicrobiae; Chthoniobacterales; Chthoniobacteraceae; LD29* |
| Mullet Gill  Tilapia Gill | Bacteria; *Fusobacteriota; Fusobacteriia; Fusobacteriales; Fusobacteriaceae; Cetobacterium* |
|  | Bacteria*; Proteobacteria; Gammaproteobacteria; Vibrionales; Vibrionaceae; Vibrio* |
|  | Bacteria; *Proteobacteria; Gammaproteobacteria; Aeromonadales; Aeromonadaceae; Aeromonas* |
|  | Bacteria; *Proteobacteria; Gammaproteobacteria; Enterobacterales; Enterobacterales unclassified; Enterobacterales unclassified* |
|  | Bacteria; *Proteobacteria; Gammaproteobacteria; Enterobacterales; Enterobacteriaceae; Plesiomonas* |
| Tilapia Skin  Mullet Skin  Water | Bacteria; *Proteobacteria; Gammaproteobacteria; Enterobacterales; Enterobacteriaceae; Enterobacteriaceae unclassified* |
|  | Bacteria; *Planctomycetota; Planctomycetes; Pirellulales; Pirellulaceae; uncultured* |
|  | Bacteria; *Verrucomicrobiota; Verrucomicrobiae; Chthoniobacterales; Chthoniobacteraceae; LD29* |
| Mullet Skin  Tilapia Skin | Bacteria; *Fusobacteriota; Fusobacteriia; Fusobacteriales; Fusobacteriaceae; Cetobacterium* |
|  | Bacteria; *Firmicutes; Bacilli; Lactobacillales; Streptococcaceae; Streptococcus* |
|  | Bacteria; *Proteobacteria; Gammaproteobacteria; Aeromonadales; Aeromonadaceae; Aeromonas* |
|  | Bacteria; *Proteobacteria; Gammaproteobacteria; Enterobacterales; Enterobacteriaceae; Plesiomonas* |
|  | Bacteria; *Proteobacteria; Gammaproteobacteria; Vibrionales; Vibrionaceae; Vibrio* |
|  | Bacteria; *Proteobacteria; Gammaproteobacteria; Pseudomonadales; Moraxellaceae; Psychrobacter* |
|  | Bacteria; *Firmicutes; Bacilli; Exiguobacterales; Exiguobacteraceae; Exiguobacterium* |
|  | Bacteria; *Proteobacteria; Gammaproteobacteria; Pseudomonadales; Moraxellaceae; Acinetobacter* |
|  | Bacteria; *Proteobacteria; Gammaproteobacteria; Burkholderiales; Chromobacteriaceae; Vogesella* |

**Supplementary Table 3. List of the core microbiota**

| Species / Tissue | Phylum | Class | Order | Family | Genus |
| --- | --- | --- | --- | --- | --- |
| Mullet / Skin | *Proteobacteria* | *Gammaproteobacteria* | *Enterobacterales* | *Enterobacteriaceae* | *Enterobacteriaceae unclassified* |
|  | *Fusobacteriota* | *Fusobacteriia* | *Fusobacteriales* | *Fusobacteriaceae* | *Cetobacterium* |
|  | *Proteobacteria* | *Gammaproteobacteria* | *Vibrionales* | *Vibrionaceae* | *Vibrio* |
|  | *Proteobacteria* | *Gammaproteobacteria* | *Aeromonadales* | *Aeromonadaceae* | *Aeromonas* |
|  | *Planctomycetota* | *Planctomycetes* | *Pirellulales* | *Pirellulaceae* | *uncultured* |
|  | *Verrucomicrobiota* | *Verrucomicrobiae* | *Chthoniobacterales* | *Chthoniobacteraceae* | *LD29* |
|  | *Firmicutes* | *Clostridia* | *Peptostreptococcales-*  *Tissierellales* | *Peptostreptococcaceae* | *Peptostreptococcaceae unclassified* |
|  | *Proteobacteria* | *Gammaproteobacteria* | *Pseudomonadales* | *Moraxellaceae* | *Psychrobacter* |
|  | *Firmicutes* | *Bacilli* | *Exiguobacterales* | *Exiguobacteraceae* | *Exiguobacterium* |
|  | *Proteobacteria* | *Gammaproteobacteria* | *Vibrionales* | *Vibrionaceae* | *Vibrionaceae unclassified* |
|  | *Bacteroidota* | *Bacteroidia* | *Bacteroidales* | *Barnesiellaceae* | *uncultured* |
|  | *Proteobacteria* | *Gammaproteobacteria* | *Pseudomonadales* | *Moraxellaceae* | *Acinetobacter* |
|  | *Proteobacteria* | *Gammaproteobacteria* | *Gammaproteobacteria unclassified* | *Gammaproteobacteria unclassified* | *Gammaproteobacteria unclassified* |
|  | *Proteobacteria* | *Gammaproteobacteria* | *Burkholderiales* | *Chromobacteriaceae* | *Vogesella* |
| Mullet / Gill | *Proteobacteria* | *Gammaproteobacteria* | *Enterobacterales* | *Enterobacteriaceae* | *Enterobacteriaceae unclassified* |
|  | *Proteobacteria* | *Gammaproteobacteria* | *Vibrionales* | *Vibrionaceae* | *Vibrio* |
|  | *Proteobacteria* | *Gammaproteobacteria* | *Aeromonadales* | *Aeromonadaceae* | *Aeromonas* |
|  | *Planctomycetota* | *Planctomycetes* | *Pirellulales* | *Pirellulaceae* | *uncultured* |
|  | *Fusobacteriota* | *Fusobacteriia* | *Fusobacteriales* | *Fusobacteriaceae* | *Cetobacterium* |
|  | *Proteobacteria* | *Gammaproteobacteria* | *Enterobacterales* | *Enterobacteriaceae* | *Plesiomonas* |
|  | *Firmicutes* | *Clostridia* | *Peptostreptococcales-Tissierellales* | *Peptostreptococcaceae* | *Peptostreptococcaceae unclassified* |
|  | *Firmicutes* | *Clostridia* | *Peptostreptococcales-Tissierellales* | *Peptostreptococcaceae* | *Paraclostridium* |
|  | *Verrucomicrobiota* | *Verrucomicrobiae* | *Chthoniobacterales* | *Chthoniobacteraceae* | *LD29* |
|  | *Firmicutes* | *Clostridia* | *Clostridiales* | *Clostridiaceae* | *Clostridiaceae unclassified* |
|  | *Firmicutes* | *Bacilli* | *Exiguobacterales* | *Exiguobacteraceae* | *Exiguobacterium* |
|  | *Actinobacteriota* | *Actinobacteria* | *PeM15* | *PeM15 fa* | *PeM15 ge* |
|  | *Proteobacteria* | *Gammaproteobacteria* | *Burkholderiales* | *Comamonadaceae* | *Comamonadaceae unclassified* |
|  | *Firmicutes* | *Clostridia* | *Clostridiales* | *Clostridiaceae* | *Clostridium sensu stricto 1* |
| Tilapia / Gill | *Proteobacteria* | *Gammaproteobacteria* | *Enterobacterales* | *Enterobacteriaceae* | *Enterobacteriaceae unclassified* |
|  | *Fusobacteriota* | *Fusobacteriia* | *Fusobacteriales* | *Fusobacteriaceae* | *Cetobacterium* |
|  | *Proteobacteria* | *Gammaproteobacteria* | *Vibrionales* | *Vibrionaceae* | *Vibrio* |
|  | *Proteobacteria* | *Gammaproteobacteria* | *Aeromonadales* | *Aeromonadaceae* | *Aeromonas* |
|  | *Planctomycetota* | *Planctomycetes* | *Pirellulales* | *Pirellulaceae* | *uncultured* |
|  | *Proteobacteria* | *Gammaproteobacteria* | *Enterobacterales* | *Enterobacteriaceae* | *Plesiomonas* |
|  | *Proteobacteria* | *Gammaproteobacteria* | *Burkholderiales* | *Burkholderiales unclassified* | *Burkholderiales unclassified* |
|  | *Verrucomicrobiota* | *Verrucomicrobiae* | *Chthoniobacterales* | *Chthoniobacteraceae* | *LD29* |
|  | *Firmicutes* | *Clostridia* | *Peptostreptococcales-Tissierellales* | *Peptostreptococcaceae* | *Peptostreptococcaceae unclassified* |
|  | *Firmicutes* | *Clostridia* | *Peptostreptococcales-Tissierellales* | *Peptostreptococcaceae* | *Paraclostridium* |
|  | *Bacteroidota* | *Bacteroidia* | *Bacteroidales* | *Barnesiellaceae* | *uncultured* |
| Tilapia / Skin | *Proteobacteria* | *Gammaproteobacteria* | *Enterobacterales* | *Enterobacteriaceae* | *Enterobacteriaceae unclassified* |
|  | *Proteobacteria* | *Gammaproteobacteria* | *Vibrionales* | *Vibrionaceae* | *Vibrio* |
|  | *Proteobacteria* | *Gammaproteobacteria* | *Aeromonadales* | *Aeromonadaceae* | *Aeromonas* |
|  | *Planctomycetota* | *Planctomycetes* | *Pirellulales* | *Pirellulaceae* | *uncultured* |
|  | *Fusobacteriota* | *Fusobacteriia* | *Fusobacteriales* | *Fusobacteriaceae* | *Cetobacterium* |
|  | *Verrucomicrobiota* | *Verrucomicrobiae* | *Chthoniobacterales* | *Chthoniobacteraceae* | *LD29* |
|  | *Firmicutes* | *Bacilli* | *Exiguobacterales* | *Exiguobacteraceae* | *Exiguobacterium* |
|  | *Firmicutes* | *Clostridia* | *Clostridiales* | *Clostridiaceae* | *Clostridium sensu stricto 1* |
|  | *Bacteroidota* | *Bacteroidia* | *Bacteroidales* | *Barnesiellaceae* | *uncultured* |
|  | *Proteobacteria* | *Gammaproteobacteria* | *Pseudomonadales* | *Moraxellaceae* | *Acinetobacter* |
| Water | *Actinobacteriota* | *Acidimicrobiia* | *Microtrichales* | *Ilumatobacteraceae* | *CL500-29 marine group* |
|  | *Actinobacteriota* | *Acidimicrobiia* | *Microtrichales* | *Ilumatobacteraceae* | *Ilumatobacteraceae unclassified* |
|  | *Actinobacteriota* | *Acidimicrobiia* | *Microtrichales* | *uncultured* | *uncultured ge* |
|  | *Actinobacteriota* | *Actinobacteria* | *Actinobacteria unclassified* | *Actinobacteria unclassified* | *Actinobacteria unclassified* |
|  | *Actinobacteriota* | *Actinobacteria* | *Frankiales* | *Sporichthyaceae* | *hgcI clade* |
|  | *Actinobacteriota* | *Actinobacteria* | *Micrococcales* | *Microbacteriaceae* | *Candidatus Aquiluna* |
|  | *Actinobacteriota* | *Actinobacteria* | *Micrococcales* | *Microbacteriaceae* | *Microbacteriaceae unclassified* |
|  | *Actinobacteriota* | *Actinobacteria* | *Nitriliruptorales* | *Nitriliruptoraceae* | *Nitriliruptoraceae ge* |
|  | *Actinobacteriota* | *Actinobacteria* | *Nitriliruptorales* | *Nitriliruptoraceae* | *Nitriliruptoraceae unclassified* |
|  | *Actinobacteriota* | *Actinobacteria* | *PeM15* | *PeM15 fa* | *PeM15 ge* |
|  | *Actinobacteriota* | *Thermoleophilia* | *Gaiellales* | *uncultured* | *uncultured ge* |
|  | *Actinobacteriota* | *Thermoleophilia* | *Solirubrobacterales* | *67-14* | *67-14 ge* |
|  | *Actinobacteriota* | *Thermoleophilia* | *Thermoleophilia unclassified* | *Thermoleophilia unclassified* | *Thermoleophilia unclassified* |
|  | *Bacteroidota* | *Bacteroidia* | *Bacteroidia unclassified* | *Bacteroidia unclassified* | *Bacteroidia unclassified* |
|  | *Bacteroidota* | *Bacteroidia* | *Chitinophagales* | *Saprospiraceae* | *Lewinella* |
|  | *Bacteroidota* | *Bacteroidia* | *Chitinophagales* | *Saprospiraceae* | *Saprospiraceae unclassified* |
|  | *Bacteroidota* | *Bacteroidia* | *Chitinophagales* | *Saprospiraceae* | *uncultured* |
|  | *Bacteroidota* | *Bacteroidia* | *Flavobacteriales* | *Crocinitomicaceae* | *Fluviicola* |
|  | *Bacteroidota* | *Kapabacteria* | *Kapabacteriales* | *Kapabacteriales fa* | *Kapabacteriales ge* |
|  | *Bacteroidota* | *Rhodothermia* | *Balneolales* | *Balneolaceae* | *Balneolaceae unclassified* |
|  | *Gemmatimonadota* | *Gemmatimonadetes* | *Gemmatimonadales* | *Gemmatimonadaceae* | *Gemmatimonas* |
|  | *Gemmatimonadota* | *Gemmatimonadetes* | *Gemmatimonadales* | *Gemmatimonadaceae* | *uncultured* |
|  | *Planctomycetota* | *Phycisphaerae* | *Phycisphaerales* | *Phycisphaeraceae* | *CL500-3* |
|  | *Planctomycetota* | *Phycisphaerae* | *Phycisphaerales* | *Phycisphaeraceae* | *Phycisphaeraceae unclassified* |
|  | *Planctomycetota* | *Planctomycetes* | *Gemmatales* | *Gemmataceae* | *uncultured* |
|  | *Planctomycetota* | *Planctomycetes* | *Isosphaerales* | *Isosphaeraceae* | *uncultured* |
|  | *Planctomycetota* | *Planctomycetes* | *Pirellulales* | *Pirellulaceae* | *Blastopirellula* |
|  | *Planctomycetota* | *Planctomycetes* | *Pirellulales* | *Pirellulaceae* | *Pirellulaceae unclassified* |
|  | *Planctomycetota* | *Planctomycetes* | *Pirellulales* | *Pirellulaceae* | *Rhodopirellula* |
|  | *Proteobacteria* | *Alphaproteobacteria* | *Alphaproteobacteria unclassified* | *Alphaproteobacteria unclassified* | *Alphaproteobacteria unclassified* |
|  | *Proteobacteria* | *Alphaproteobacteria* | *Rhodobacterales* | *Rhodobacteraceae* | *Rhodobacter* |
|  | *Proteobacteria* | *Alphaproteobacteria* | *Rhodobacterales* | *Rhodobacteraceae* | *Rhodobacteraceae unclassified* |
|  | *Proteobacteria* | *Alphaproteobacteria* | *Sphingomonadales* | *Sphingomonadaceae* | *Rhizorhapis* |
|  | *Proteobacteria* | *Gammaproteobacteria* | *Burkholderiales* | *Burkholderiaceae* | *Burkholderiaceae unclassified* |
|  | *Proteobacteria* | *Gammaproteobacteria* | *Burkholderiales* | *Burkholderiaceae* | *Polynucleobacter* |
|  | *Proteobacteria* | *Gammaproteobacteria* | *Burkholderiales* | *Comamonadaceae* | *Comamonadaceae unclassified* |
|  | *Proteobacteria* | *Gammaproteobacteria* | *Burkholderiales* | *Comamonadaceae* | *Rubrivivax* |
|  | *Proteobacteria* | *Gammaproteobacteria* | *Burkholderiales* | *MWH-UniP1 aquatic group* | *MWH-UniP1 aquatic group ge* |
|  | *Proteobacteria* | *Gammaproteobacteria* | *Cellvibrionales* | *Cellvibrionaceae* | *Cellvibrio* |
|  | *Proteobacteria* | *Gammaproteobacteria* | *Enterobacterales* | *Enterobacteriaceae* | *Enterobacteriaceae unclassified* |
|  | *Proteobacteria* | *Gammaproteobacteria* | *Gammaproteobacteria unclassified* | *Gammaproteobacteria unclassified* | *Gammaproteobacteria unclassified* |
|  | *Proteobacteria* | *Gammaproteobacteria* | *JG36-TzT-191* | *JG36-TzT-191 fa* | *JG36-TzT-191 ge* |
|  | *Proteobacteria* | *Gammaproteobacteria* | *Vibrionales* | *Vibrionaceae* | *Vibrio* |
|  | *Verrucomicrobiota* | *Chlamydiae* | *Chlamydiales* | *Chlamydiales unclassified* | *Chlamydiales unclassified* |
|  | *Verrucomicrobiota* | *Verrucomicrobiae* | *Chthoniobacterales* | *Chthoniobacteraceae* | *LD29* |
|  | *Verrucomicrobiota* | *Verrucomicrobiae* | *Chthoniobacterales* | *Terrimicrobiaceae* | *Terrimicrobium* |
