# Supplementary material for: Characterization of External Mucosal Microbiomes of Nile Tilapia and Grey Mullet Co-cultured in Semi-Intensive Pond Systems
Source: Front Microbiol. 2021 Dec 13;12:773860. doi: 10.3389/fmicb.2021.773860 (PMC8710667; doi:10.3389/fmicb.2021.773860)
Supplement: Supplementary file 5 [file Data_Sheet_4.pdf]

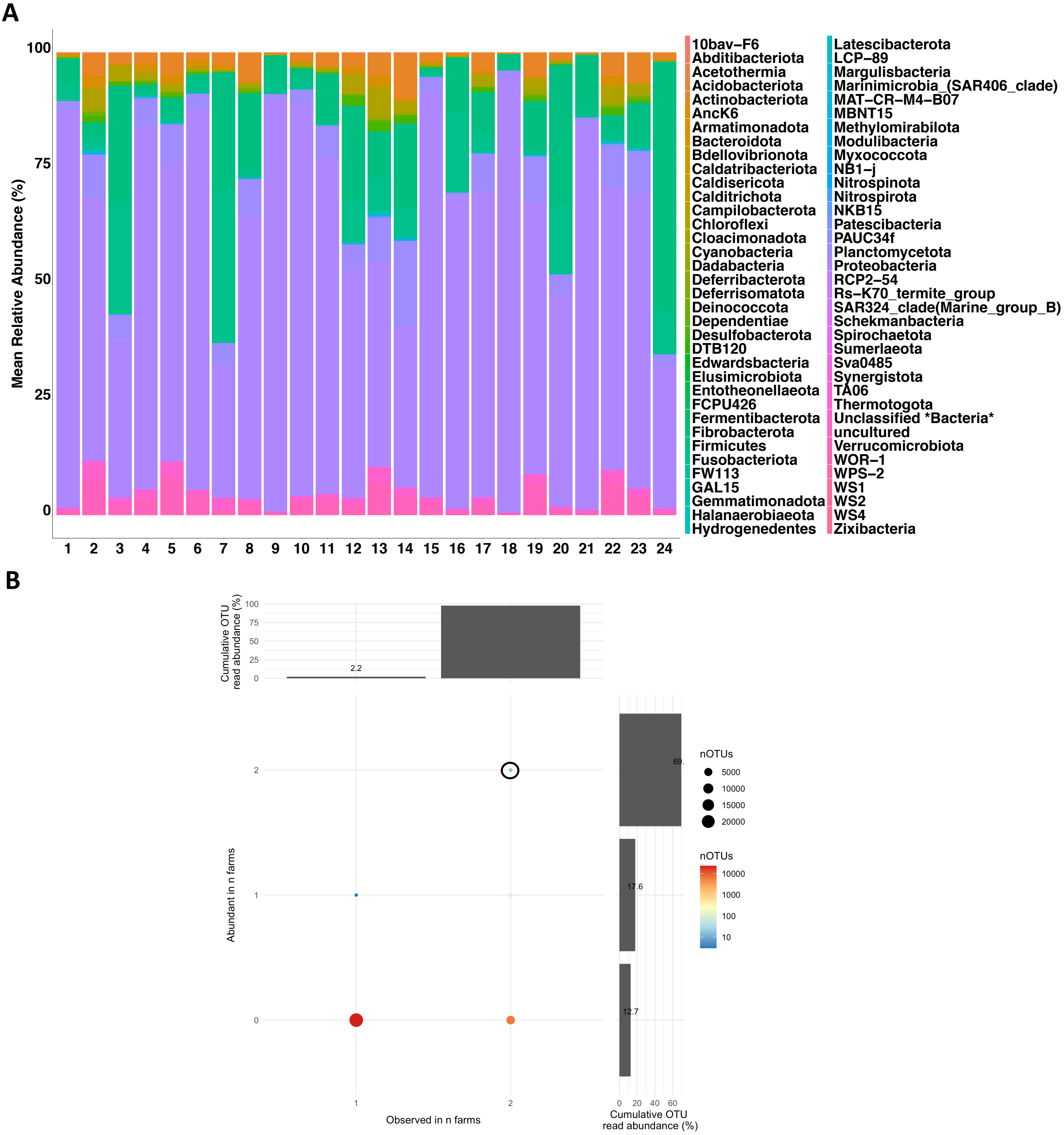

Supplementary Figure 4 Mean relative abundance (%) of the overall prevalent phyla and core microbiome in grey mullet gill (n= 24 fish) across two farms. **(A)** Mean relative abundance at phylum level. **(B)** Distribution of OTUs-level taxa of mullet gill microbiome across the farms. OTUs are grouped based on the number of farms in which they are observed (x axis) and number of farms in which they are abundant (y axis). Number of OTUs belonging to each group is represented by colour and circle size. The core community is defined as present and abundant in all farms analysed and is marked with black, empty circle. Cumulative abundance of OTUs observed in n farms and abundant in n farms are given in upper and right margin plot, respectively.
